# Influence of dentin depth and hemostatic contamination on microtensile bond strength of a universal adhesive: Effect of phosphoric acid re-etching as a rescue protocol

**DOI:** 10.4317/jced.64286

**Published:** 2026-08-28

**Authors:** Gaurav Patri, Debkant Jena, Prasanti Kumari Pradhan, Pratik Agrawal, Akansha Tilokani, Swadheena Patro

**Affiliations:** 1 Department of Conservative Dentistry and Endodontics, Kalinga Institute of Dental Sciences (KIDS), KIIT Deemed to be University, Bhubaneswar, 751024, Odisha, India

**Keywords:** Aluminum chloride, dentin bonding agents, hemostatic agents, microtensile bond strength, phosphoric acids, universal adhesive.

## Abstract

**Background:**

This study evaluated the influence of dentin depth and surface treatment on the microtensile bond strength (µTBS) of a universal adhesive to dentin contaminated with an aluminum chloride hemostatic agent, and assessed the effectiveness of phosphoric acid re-etching as a contamination management strategy.

**Materials and Methods:**

Sixty extracted human third molars were assigned to six groups (n=10) based on dentin depth (superficial or deep) and surface treatment (control, contamination, and contamination followed by phosphoric acid re-etching). A universal adhesive was applied in self-etch mode for control and contamination groups, and in etch-and-rinse mode following the rescue protocol. Composite build-up was performed and specimens were thermocycled (10,000 cycles). µTBS was measured using a universal testing machine. Data were analyzed using two-way ANOVA and Tukey’s post hoc test (p<0.05). Failure modes were evaluated using stereomicroscopy and analyzed with Pearson’s chi-square test.

**Results:**

Superficial dentin demonstrated significantly higher µTBS than deep dentin (p<0.001). Aluminum chloride contamination significantly reduced bond strength in both substrates (p<0.05). Phosphoric acid re-etching significantly improved bond strength in superficial dentin (p<0.05) but did not restore control values. In deep dentin, the rescue protocol did not significantly improve bond strength (p>0.05). A significant interaction between dentin depth and surface treatment was observed (p=0.001).

**Conclusions:**

Dentin depth significantly influences the effectiveness of contamination management strategies. Phosphoric acid re-etching partially restores bond strength in superficial dentin but is ineffective in deep dentin, indicating that contamination management is substrate-dependent.

## Introduction

Dentin, with its structural diversity, plays an important role in determining adhesive bond strength [1]. The substrate available for micromechanical interlocking depends on the amount of intertubular dentin and the density of dentinal tubules in any given region-areas with more intertubular dentin tend to produce higher bond strength values. These structural differences directly shape how adhesive systems interact with dentin and, by extension, the overall effectiveness of the bonded interface [1].

It is well established that superficial dentin yields higher bond strength than deep dentin [2,3]. This difference is largely driven by changes in dentin morphology with increasing depth: Greater tubule density, wider tubule diameters, and more pronounced dentinal fluid movement associated with pulpal pressure [4]. Each of these factors can interfere with adhesive infiltration and polymerization, ultimately compromising the quality and stability of the bonded layer [4]. Even modern adhesive systems are not exempt from this substrate-dependent variability [5].

On top of intrinsic substrate differences, extrinsic contamination can substantially undermine dentin adhesion [6]. Hemostasis is one of the most common sources of contamination in clinical practice, and aluminum chloride-based agents are routinely used to control bleeding and crevicular fluid seepage during tooth preparation. Their residual deposits alter the dentin surface and can occlude dentinal tubules, interfering with adhesive interaction and reducing bond strength [6-9]. This effect is particularly pronounced in self-etch adhesive systems, which rely on simultaneous demineralization and resin infiltration [6]. Universal adhesives used in self-etch mode also remain susceptible to this effect after aging [7-9].

Because contamination is clinically difficult to avoid entirely, decontamination strategies matter. Phosphoric acid etching has been widely studied for its ability to remove surface contaminants and modify the smear layer [10-12]. Its effectiveness, however, is not straightforward; it varies with the adhesive system, the application protocol, and the clinical context [10-12]. Importantly, applying phosphoric acid after contamination inherently converts a self-etch bonding approach into an etch-and-rinse one, which introduces an additional variable when interpreting bond strength outcomes [10-12]. Some studies report that this strategy can improve bonding after contamination [11], while others show only partial or inconsistent recovery [10,12]. Phosphoric acid appears to outperform water rinsing, air abrasion, and proprietary cleaning agents [12,13], and the broader literature confirms that both contamination and decontamination techniques meaningfully affect adhesive performance [14].

What remains less well understood is how dentin depth interacts with hemostatic contamination and subsequent decontamination. These two variables have mostly been studied in isolation [1-5,6-9]. Studies evaluating decontamination protocols have generally used standardized superficial dentin to limit variability, without considering depth as a potentially modifying factor [7,8,12]. This is a meaningful gap; in deep cavity preparations, where hemostatic agents are perhaps most commonly needed, the substrate is already compromised by structural and hydrodynamic factors. Whether phosphoric acid re-etching can restore bond strength consistently across different dentin depths is, at present, unclear. To the authors' knowledge, the interaction between dentin depth and phosphoric acid rescue following aluminum chloride contamination has received limited investigation.

This study was therefore designed to examine the interaction between dentin depth and surface treatment on the bond strength of a universal adhesive. The specific aim was to determine whether dentin depth influences the effectiveness of phosphoric acid re-etching as a rescue protocol following aluminum chloride contamination. Three null hypotheses were tested: That dentin depth would not affect bond strength, that surface treatment would not influence bond strength, and that no significant interaction would exist between these two variables.

## Materials and Methods

*Study design*

This *in vitro* experimental study evaluated the effects of dentin depth and surface treatment on the microtensile bond strength (µTBS) of a universal adhesive bonded to dentin contaminated with an aluminum chloride hemostatic agent, in accordance with Academy of Dental Materials recommendations [15]. Ethical approval was obtained from the Institutional Ethics Committee (Ref. No. KIIT/KIMS/IEC/1236/2025), and all procedures were conducted in accordance with the Declaration of Helsinki.

The independent variables were dentin depth (superficial and deep) and surface treatment (control, contamination, and contamination followed by phosphoric acid re-etching). The dependent variable was µTBS in MPa.

The phosphoric acid rescue protocol in this study inherently converts the adhesive application from self-etch to etch-and-rinse mode. This was a deliberate decision; the aim was to replicate what a clinician would realistically do when encountering contamination, rather than to isolate the effect of phosphoric acid independently of bonding strategy. The change in adhesive mode is therefore a feature of the rescue protocol, not a confound to be corrected. That said, it does mean that any bond strength improvement observed in the rescue groups reflects the combined effect of contaminant removal and the shift to etch-and-rinse mode, and these two contributions cannot be separated within this study design. This aspect should be considered when interpreting the findings.

*Sample size estimation*

Sample size was calculated using G*Power software (v3.1; Heinrich Heine University, Düsseldorf, Germany) for a two-way ANOVA, based on effect sizes from previous studies evaluating hemostatic contamination effects on dentin bond strength [3,7,8]. Effect size (f) was derived using the formula f=√(η² / (1-η²). A large effect size of 0.40 was selected, consistent with prior reports of substantial bond strength reductions following hemostatic agent contamination [3,7,8]. With α=0.05, statistical power of 80%, and six experimental groups, a minimum of 10 teeth per group was required (total n=60) [15]. The tooth was used as the statistical unit, with mean µTBS per tooth calculated to avoid pseudo-replication [15].

*Tooth selection and storage*

Sixty extracted, non-carious, unrestored human third molars were collected under ethical approval and with informed consent. Teeth with caries, restorations, cracks, or visible structural defects were excluded. After removing adhering soft tissue debris, all teeth were stored in 0.1% thymol solution at 4°C for a maximum of three months, then rinsed under running water for 24 hours before specimen preparation to remove residual storage medium.

*Preparation of superficial and deep dentin*

Teeth were embedded in self-curing acrylic resin for standardized handling. Occlusal enamel was removed using a water-cooled low-speed diamond saw (Isomet; Buehler Ltd., Lake Bluff, IL, USA) to expose the underlying dentin. Superficial dentin was defined as dentin immediately beneath the deepest occlusal groove; deep dentin was obtained by removing an additional 2.0 mm from the superficial surface (Figure 1 A,B) [2,3].

Dentin depth was verified with a digital caliper (accuracy  ± 0.01 mm; Corceptive, India), and any specimen with pulpal exposure was excluded [3]. All surfaces were standardized by wet polishing with 600-grit silicon carbide paper (3M India Ltd., India) for 60 seconds to create a uniform smear layer [15], a protocol accepted as simulating clinically produced smear layers [15].

*Experimental group allocation*

Teeth were randomly allocated to six groups (n=10 per group) using a computer-generated randomization sequence (Random.org, Dublin, Ireland). Allocation was managed by an independent operator not involved in specimen preparation or testing. All laboratory procedures were carried out by a single calibrated operator. Group composition and treatment protocols are summarized in Table 1.

*Hemostatic agent contamination protocol*

Dentin surfaces in the contamination groups were treated with a hemostatic agent (ViscoStat Clear; 25% aluminum chloride; Ultradent Products Inc., South Jordan, UT, USA) applied using a microbrush for 60 seconds. Specimens were then rinsed with water for 30 seconds and gently air-dried for 5 seconds, leaving the dentin visibly moist without pooling [7,9,11].

*Phosphoric acid rescue protocol*

In the rescue groups, contaminated dentin surfaces were treated with 37% phosphoric acid (Scotchbond Multipurpose Etchant, 3M ESPE, USA) for 15 seconds, rinsed thoroughly for 15 seconds, and blot-dried to achieve a moist dentin surface. Adhesive application then followed the etch-and-rinse approach, as described below [10-12].

*Adhesive application*

A universal adhesive (Single Bond Universal, 3M ESPE, USA) was applied following manufacturer instructions. For control and contamination groups (self-etch mode), the adhesive was actively scrubbed onto the dentin surface for 20 seconds, then gently air-thinned for 5 seconds to evaporate the solvent, and light-cured for 10 seconds [7,9]. For rescue groups, adhesive was applied after phosphoric acid pretreatment in etch-and-rinse mode [11]. Light curing was performed with an LED unit (Bluephase N, Ivoclar Vivadent, Schaan, Liechtenstein; emission range 380-515 nm, output ≥1000mW/cm²), with intensity verified periodically using a calibrated radiometer.

*Composite resin build-up*

A nanohybrid composite resin (Filtek Z350 XT, 3M ESPE, USA) was built up incrementally in 2- mm layers, each light-cured for 20 seconds, to a final height of approximately 4-5 mm (Figure 1C) [15].

*Aging procedure*

Specimens were stored in distilled water at 37°C for 24 hours, then thermocycled for 10,000 cycles between 5°C and 55°C (dwell time 30 seconds, transfer time 5 seconds; SD Mechatronik GmbH, Feldkirchen-Westerham, Germany), approximating one year of clinical aging per established protocols [15,16].

*Microtensile specimen preparation*

Each tooth was sectioned longitudinally in both mesiodistal and buccolingual planes using a water-cooled diamond saw (Isomet; Buehler Ltd.) to obtain resin-dentin beams with a cross-sectional area of 2.0 ± 0.1 mm². Dimensions were verified with a digital caliper; beams with irregular geometry were excluded. A mean of 7 ± 1 beams was obtained per tooth. The mean µTBS per tooth was used as the statistical unit throughout to avoid pseudo-replication. No pretest failures were recorded.

*Microtensile bond strength testing*

Each beam was attached to a microtensile testing jig and loaded to failure in a universal testing machine (ZwickRoell GmbH & Co. KG, Ulm, Germany) at a crosshead speed of 1 mm/min. µTBS (MPa) was calculated by dividing maximum load at failure (N) by cross-sectional area (mm²) [15].

*Failure mode analysis*

Fractured specimens were examined under a stereomicroscope (Leica Microsystems, Wetzlar, Germany) at 40× magnification and classified as adhesive, cohesive, or mixed failures (Figure 2) [3,7,11,15]. 

The examiner performing the evaluation was blinded to group allocation to minimize assessment bias.

*Statistical analysis*

Statistical analysis was performed using SPSS version 26.0 (IBM Corp., Armonk, NY, USA). Data normality and homogeneity of variance were assessed with the Shapiro-Wilk and Levene's tests, respectively. A two-way ANOVA was used to evaluate the effects of dentin depth and surface treatment on µTBS, followed by Tukey's post hoc test for pairwise comparisons. Effect sizes (partial η²) were calculated. Failure mode distributions were compared using Pearson's chi-square test. Statistical significance was set at *p<*0.05.

## Results

Mean µTBS values for all experimental groups are presented in Table 2.

In superficial dentin, aluminum chloride contamination reduced bond strength relative to the control group (18.27 ± 2.62 MPa vs. 30.60 ± 3.08 MPa; *p<*0.05). Following the phosphoric acid rescue protocol, bond strength increased to 27.45 ± 2.50 MPa, a statistically significant improvement over the contaminated group (*p<*0.05), though values remained below those of the uncontaminated control (*p<*0.05). In deep dentin, contamination similarly reduced bond strength compared with the control group (15.80 ± 2.60 MPa vs. 26.00 ± 2.92 MPa; *p<*0.05). Application of the rescue protocol yielded 18.36 ± 2.50 MPa, a difference from the contaminated group that did not reach statistical significance (*p>*0.05), and values remained significantly below the deep dentin control (*p<*0.05).

Two-way ANOVA results are presented in Table 3. 

Significant main effects were observed for both dentin depth (F=59.26, *p<*0.001, partial η²=0.523) and surface treatment (F=86.41, *p<*0.001, partial η²=0.762). A significant interaction between the two factors was also detected (F=7.78, *p=*0.001, partial η²=0.224), indicating that the effect of surface treatment on bond strength was not consistent across dentin depths. This interaction is illustrated in Figure 3.

Failure mode distributions are shown in Figure 4. A statistically significant association between experimental group and failure pattern was found (χ²=22.60, df=10, *p=*0.012). In superficial control specimens, failures were predominantly mixed (60%) and cohesive in dentin (30%), with 10% adhesive failures. Contamination shifted this distribution markedly toward adhesive failures: 70% in superficial dentin and 80% in deep dentin. Following the phosphoric acid rescue protocol, superficial dentin specimens showed a reduction in adhesive failures to 20%, with a corresponding increase in mixed failures to 60% and cohesive failures in dentin to 20%. In deep dentin rescue specimens, adhesive failures remained predominant (60%), cohesive failures in resin accounted for 20%, mixed failures for 20%, and no cohesive failures in dentin were recorded.

## Discussion

This study was motivated by a gap between laboratory practice and clinical reality. Hemostatic agents are most commonly deployed during deep cavity preparations - precisely the setting where adhesive bonding is already under substrate-related stress - yet the experimental literature on hemostatic contamination has relied almost entirely on superficial, standardized dentin surfaces. By crossing two levels of dentin depth with hemostatic contamination and a phosphoric acid rescue protocol, this study asked whether depth modifies the outcome of decontamination. The answer is yes. All three null hypotheses were rejected, and the significant depth-treatment interaction (*p=*0.001) is the core finding: The capacity of phosphoric acid re-etching to rescue bond strength after aluminum chloride contamination depends substantially on the depth of the substrate to which it is applied.

Previous investigations have largely evaluated hemostatic contamination using standardized superficial dentin surfaces and have focused primarily on the effectiveness of various cleaning procedures [7,10-12]. In contrast, the present study examined whether dentin depth modifies the response to a commonly employed rescue strategy, thereby introducing a clinically important substrate-related variable that has received limited attention.

That bond strength was higher in superficial dentin than in deep dentin was expected and consistent with established reports [2,3]. The structural basis for this difference is well characterized. Moving deeper into dentin, the proportion of intertubular dentin - the solid matrix that supports micromechanical interlocking - progressively diminishes, while tubule density and diameter increase [1-3]. Increased dentinal permeability at greater depths drives outward fluid movement that disrupts adhesive infiltration and impairs monomer polymerization at the bonding interface [4]. Taken together, these structural and hydrodynamic factors produce a bonding substrate that is already at a disadvantage before any contamination is introduced - and this baseline difference is evident in the present data.  These findings align with previous studies reporting superior bonding performance in superficial dentin for both etch-and-rinse and self-etch systems [2,3]. In self-etch adhesives, bonding is strongly governed by monomer interaction with the smear layer and the depth of demineralization relative to resin infiltration [17]. The long-term performance of universal adhesives is similarly dependent on the quality of adhesive infiltration into dentin [18]. Effective bonding therefore requires adequate monomer penetration and stable hybrid layer formation [19-21], processes that are inherently less reliable in deep dentin given its altered substrate characteristics.

Aluminum chloride contamination reduced bond strength in both substrates, findings that sit comfortably within a consistent body of published evidence [6-9,22]. The mechanism is reasonably well understood: Residual aluminum deposits occlude dentinal tubules and alter surface chemistry, both of which limit adhesive penetration and weaken the bonded interface [6-9,22]. Self-etch systems are particularly vulnerable because their simultaneous demineralization-and-infiltration mechanism requires direct contact with a clean, reactive smear layer; something the hemostatic agent effectively disrupts [6]. The magnitude of reduction observed here is broadly consistent with what has been reported for universal adhesives used in self-etch mode under similar contamination conditions [7-9]. Systematic evidence further corroborates that hemostatic agents impair bonding by altering dentin surface characteristics and reducing adhesive-substrate interaction [6,14].

In superficial dentin, the rescue protocol improved bond strength, though not to control levels; recovery was real but incomplete. This partial restoration is consistent with prior reports [10,14] and most plausibly reflects two concurrent effects: Removal of surface contaminants (aluminum residues cleared, occluded tubules reopened) and the shift from self-etch to etch-and-rinse bonding mode [10-12,22,23]. The ability of phosphoric acid to remove contaminants and modify the dentin surface enhances adhesive interaction and infiltration, which likely underpins the observed improvement [10-13,17]. Separating these contributions is not possible within the current design - a group receiving phosphoric acid on uncontaminated dentin would have been necessary for that purpose. That group was deliberately omitted, since the study was built around a clinical scenario in which etching would only occur as a corrective response to contamination, not as a routine step. What the data make clear, however, is that the combined intervention, phosphoric acid application plus etch-and-rinse adhesive placement, still falls short of restoring the original interface, suggesting that some degree of surface alteration from aluminum chloride persists despite treatment [6,10,14]. Consequently, the observed improvement should be interpreted as the effect of the entire rescue protocol rather than phosphoric acid treatment alone.

Deep dentin tells a different story. The rescue protocol produced no statistically significant improvement over the contaminated group; a finding with direct clinical relevance, since deep cavity preparations are precisely the settings where hemostatic agents are most frequently needed. What this result implies is that in the substrate most likely to be contaminated in practice, the most widely recommended decontamination approach may offer little meaningful benefit. The structural constraints of deep dentin appear to set a ceiling on what any surface-directed intervention can accomplish. Even when contaminants are removed, the reduced intertubular dentin and the heightened fluid dynamics at the interface continue to impede adhesive infiltration and hybrid layer formation [1-4]. There is an additional problem specific to etch-and-rinse bonding in fluid-rich substrates: Phosphoric acid demineralizes more aggressively than resin monomers can infiltrate, leaving exposed collagen that is incompletely supported and mechanically fragile [17-20]. This mismatch between demineralization depth and resin penetration depth is well recognized in the wet bonding literature, and deep dentin, which is wetter and more hydraulically active than superficial dentin [4], likely amplifies it further.

The failure mode distributions support and extend the bond strength findings. In uncontaminated specimens, failures were predominantly mixed or cohesive in dentin - a pattern generally indicative of a reasonably robust adhesive interface [3,7,11,15]. Contamination shifted this distribution heavily toward adhesive failures in both substrates, which is consistent with interfacial disruption rather than bulk material weakness [7-9]. The response to rescue treatment diverged markedly between depths. In superficial dentin, the distribution shifted back toward mixed failures after phosphoric acid treatment; a pattern associated with partial but genuine interface recovery [10-13,23]. In deep dentin rescue specimens, adhesive failures remained predominant, reinforcing the conclusion that surface decontamination alone cannot overcome the bonding limitations imposed by the substrate itself.

From a clinical perspective, the findings suggest that phosphoric acid re-etching may serve as a useful rescue strategy following aluminum chloride contamination in shallow cavity preparations. However, in deep dentin, where substrate characteristics are inherently less favorable, prevention of contamination appears to be more predictable than attempting bond recovery after contamination has occurred.

Several limitations deserve acknowledgement. As an *in vitro* study, it cannot replicate the full clinical environment-salivary contamination, operator variability, the timing of hemostatic agent application relative to adhesive placement, and *in vivo* pulpal pressure are all absent from this model. Blood contamination was also not reproduced in the present model, despite its frequent coexistence with hemostatic agent application under clinical conditions. Only one universal adhesive and one aluminum chloride hemostatic agent were evaluated; therefore, extrapolation of these findings to other adhesive systems and hemostatic formulations should be performed with caution. The conversion from self-etch to etch-and-rinse mode in the rescue groups remains the most important interpretive caveat: Because the two effects (contaminant removal and bonding mode change) are inseparable in this design, clinicians should be cautious about attributing observed improvements entirely to decontamination. Long-term mechanical degradation under oral conditions was not assessed, though thermocycling was included as a proxy for early aging.

Further studies should test a wider range of universal adhesives and hemostatic agents, and should investigate decontamination approaches that do not require a change in bonding mode; which would allow the cleaning effect to be isolated from the adhesive strategy effect. Interfacial characterization using scanning electron or transmission electron microscopy would also be informative, since it would help distinguish between a surface-cleaning failure and an infiltration failure in deep dentin rescue specimens - two mechanistically distinct problems that require different solutions. Further clinical studies are required to determine whether the substrate-dependent effects observed *in vitro* translate into long-term clinical performance.

## Conclusions

Dentin depth significantly influences both adhesive bond strength and the effectiveness of contamination management. Aluminum chloride contamination reduces bonding in superficial and deep dentin alike. Phosphoric acid re-etching partially improves bond strength in superficial dentin but does not restore uncontaminated values, and it offers no significant benefit in deep dentin. These findings indicate that contamination management strategies are substrate-dependent and cannot be applied uniformly across different dentin depths. Clinically, this underlines the importance of strict contamination prevention, particularly in deep dentin, where the window for effective corrective intervention appears to be narrow. 

## Figures and Tables

**Table 1:** Experimental groups and surface treatment protocols.

**Group**	**Dentin Depth**	**Surface Treatment Protocol**	**Adhesive Mode**
G1	Superficial	No hemostatic agent; no decontamination (control)	Self-etch
G2	Superficial	ViscoStat Clearᵃ (25% AlCl₃), 60 s → water rinse 30 s	Self-etch
G3	Superficial	ViscoStat Clearᵃ (25% AlCl₃), 60 s → 37% H₃PO₄ᵇ, 15 s → water rinse 15 s → blot-dry	Etch-and-rinse (rescue)
G4	Deep	No hemostatic agent; no decontamination (control)	Self-etch
G5	Deep	ViscoStat Clearᵃ (25% AlCl₃), 60 s → water rinse 30 s	Self-etch
G6	Deep	ViscoStat Clearᵃ (25% AlCl₃), 60 s → 37% H₃PO₄ᵇ, 15 s → water rinse 15 s → blot-dry	Etch-and-rinse (rescue)

All groups received Single Bond Universal adhesive (3M ESPE, St. Paul, MN, USA). ^a^ Ultradent Products Inc., South Jordan, UT, USA. ^b^ Scotchbond Multipurpose Etchant, 3M ESPE, USA. AlCl_3_, aluminum chloride; H_3_PO_4_, phosphoric acid.

**Table 2:** Microtensile bond strength (µTBS) according to dentin depth and surface treatment.

**Dentin Depth**	**Surface Treatment**	**n**	**µTBS (MPa), mean ± SD**
Superficial	Control	10	**30.60 ± 3.08** ^a^
Superficial	Contaminated	10	18.27 ± 2.62^c^
Superficial	Rescue	10	27.45 ± 2.50^b^
Deep	Control	10	26.00 ± 2.92^b^
Deep	Contaminated	10	15.80 ± 2.60^c^
Deep	Rescue	10	18.36 ± 2.50^c^

Values expressed as mean ± standard deviation. µTBS, microtensile bond strength. Different superscript letters indicate statistically significant differences (Tukey's post hoc test, α=0.05). Groups sharing a letter are not significantly different.

**Table 3:** Two-way ANOVA results for the effects of dentin depth and surface treatment on µTBS.

**Source of Variation**	**df**	*F-value*	*p-value*	**Partial η²**
Dentin depth	1	59.26	<0.001	0.523
Surface treatment	2	86.41	<0.001	0.762
Depth × Treatment	2	7.78	0.001	0.224
Error	54	-	-	-

Two-way ANOVA followed by Tukey's post hoc test (α=0.05). Partial η^2^ represents effect size. A significant interaction indicates that the effect of surface treatment varies according to dentin depth.

**Figure 1:**
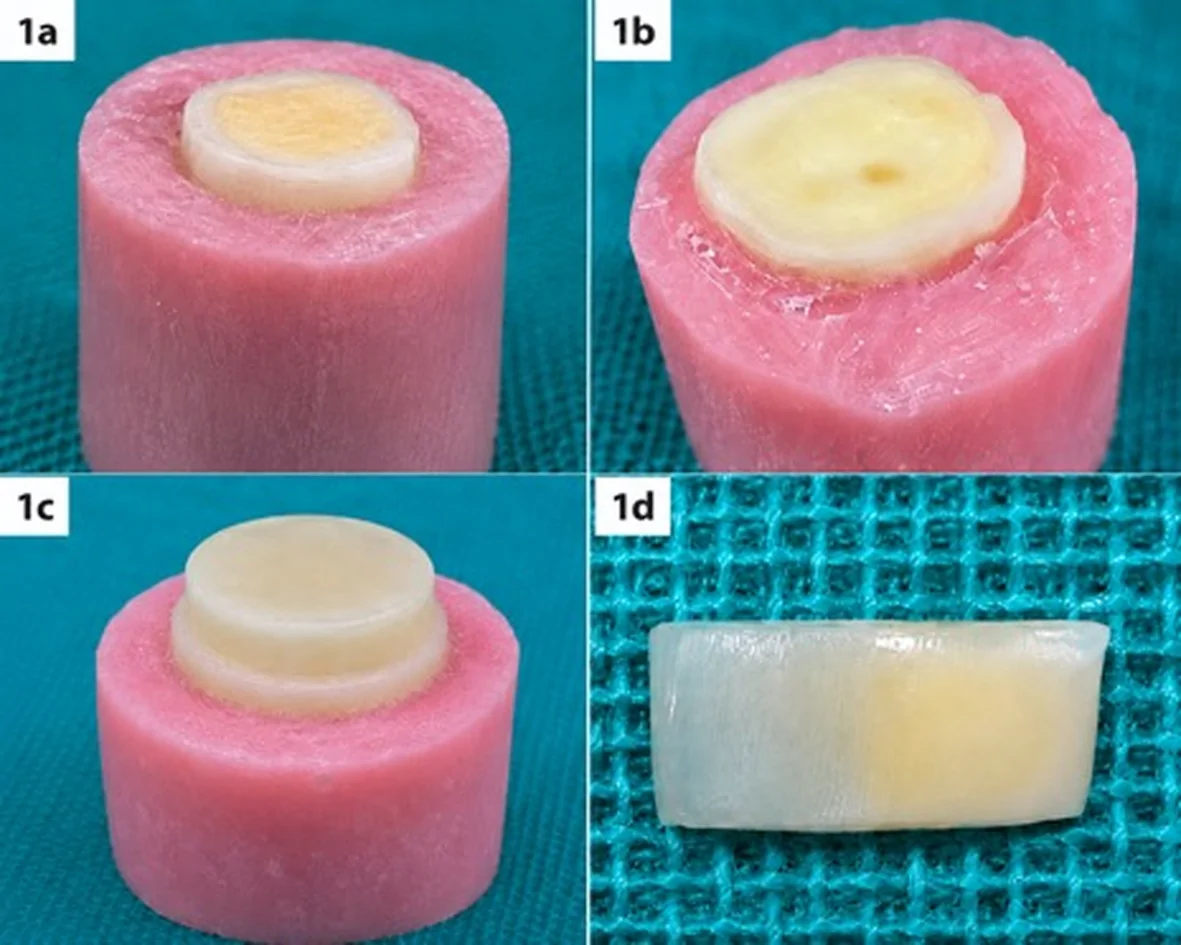
Representative images of dentin specimen preparation and composite build-up. A: Superficial dentin following occlusal enamel removal; B: Deep dentin after additional 2.0-mm dentin reduction; C: Incremental composite resin build-up; D: Sectioned resin-dentin beams prepared for µTBS testing.

**Figure 2:**
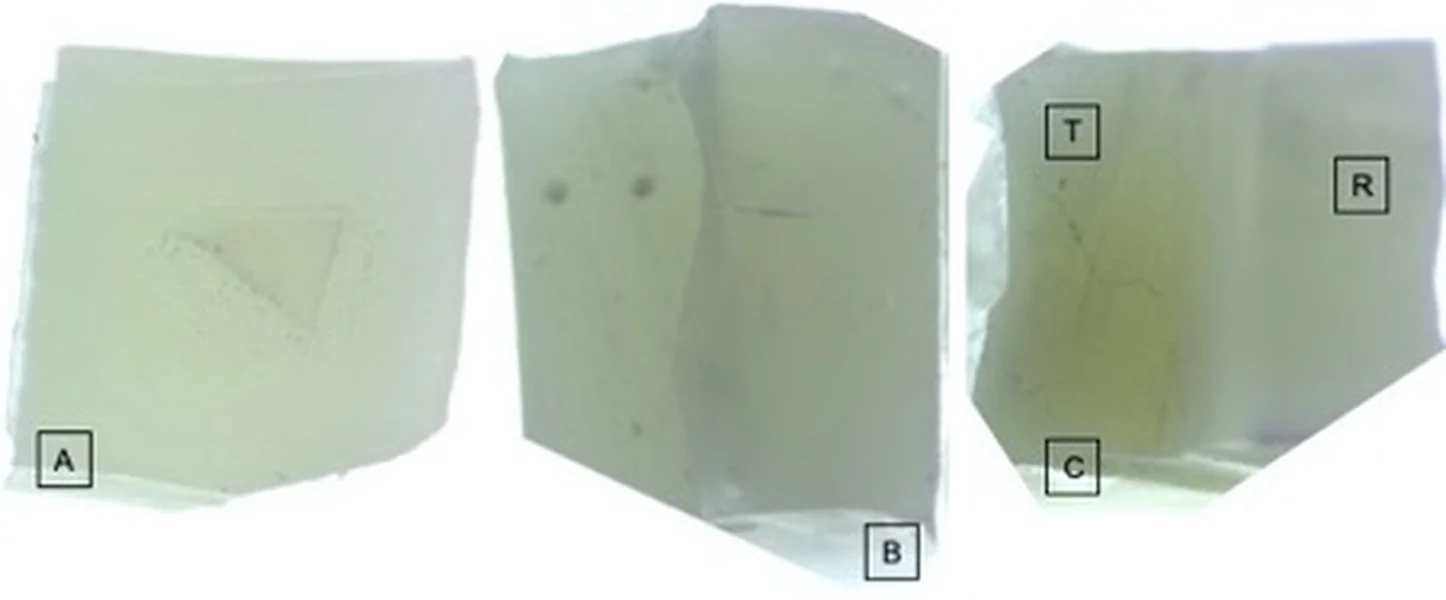
Representative stereomicroscopic images (40× magnification) of failure modes following microtensile bond strength (µTBS) testing. A: Adhesive failure; B: Cohesive failure; and C: Mixed failure. T denotes dentin and R denotes resin composite.

**Figure 3:**
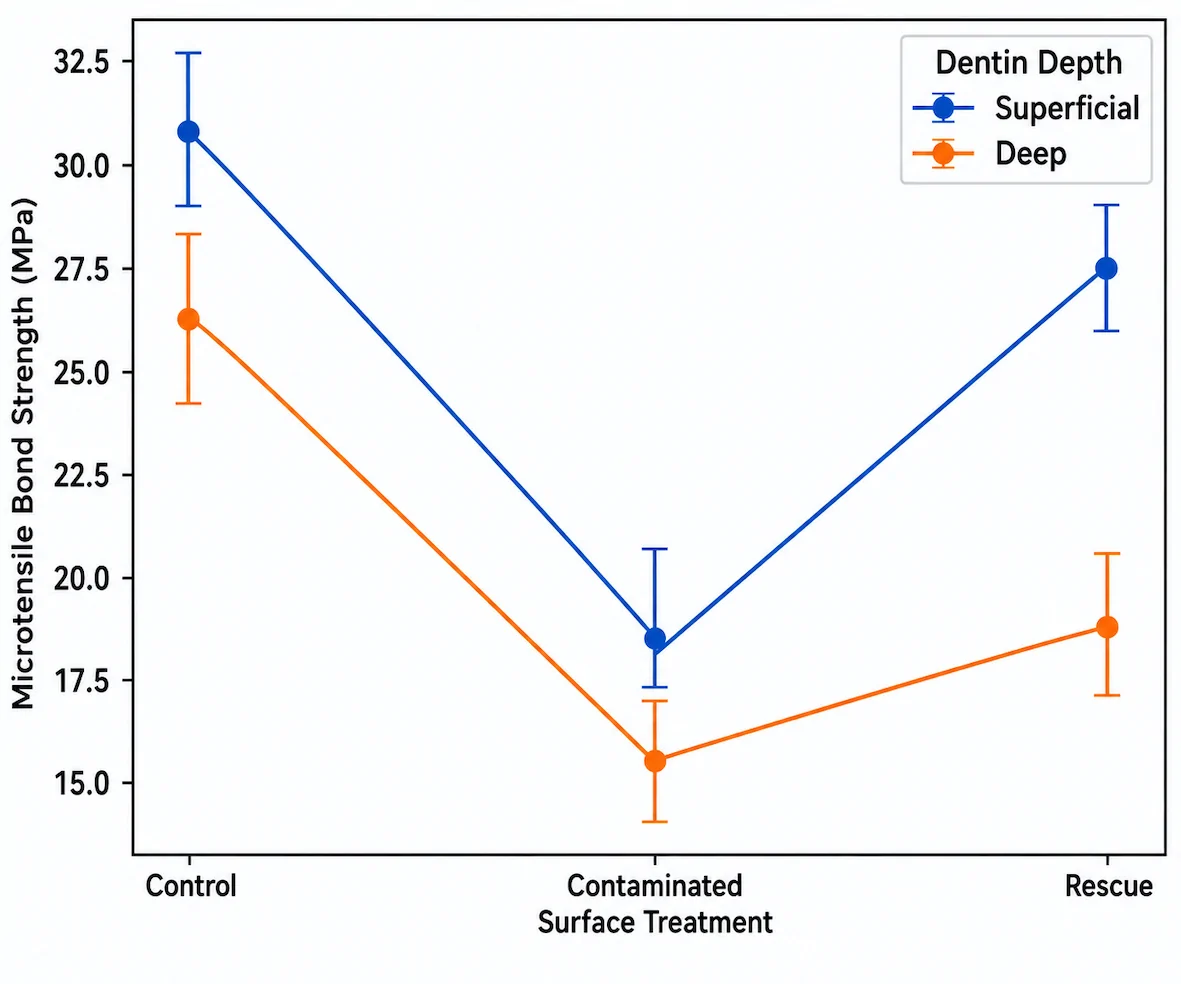
Interaction plot showing mean µTBS (± SD) as a function of dentin depth and surface treatment (n=10 per group). A significant Depth × Treatment interaction was observed (two-way ANOVA, p=0.001), reflecting greater bond strength recovery following the rescue protocol in superficial dentin compared with deep dentin.

**Figure 4:**
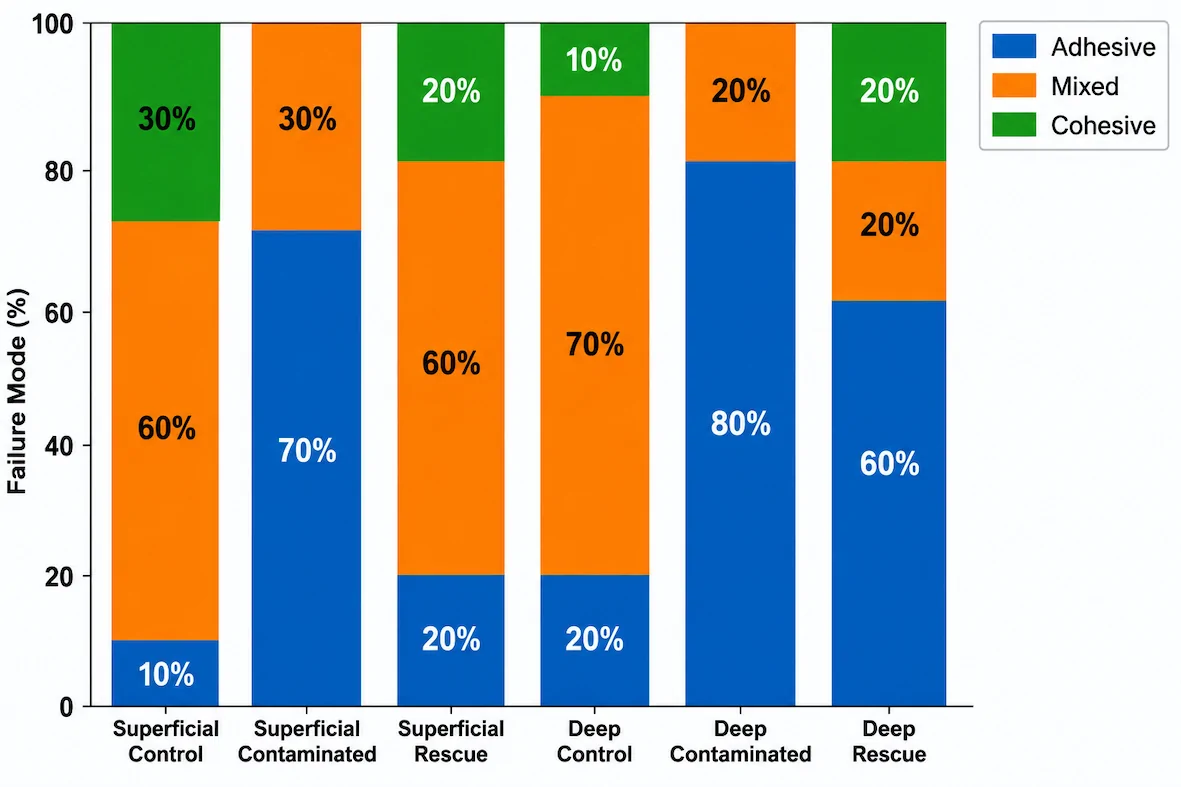
Failure mode distribution across experimental groups. Modes classified as adhesive, cohesive in dentin, cohesive in resin, and mixed following stereomicroscopic examination (40×). Percentages represent within-group distribution (n=10 per group). A statistically significant association was found between group and failure pattern (χ^2^=22.60, df=10, p=0.012).

## Data Availability

Declared none.
